# Community pharmacy staff’s response to symptoms of common infections: a pseudo-patient study

**DOI:** 10.1186/s13756-019-0510-x

**Published:** 2019-03-29

**Authors:** Shukry Zawahir, Sarath Lekamwasam, Parisa Aslani

**Affiliations:** 1 The University of Sydney School of Pharmacy, Sydney, NSW Australia; 2 Population Health Research Centre, Department of Medicine, Faculty of Medicine, University of Ruhuna, Galle, Sri Lanka

**Keywords:** Antibiotic, Antibiotic resistance, Community pharmacy, Dispensing, Pharmacy staff, Sri Lanka, Pseudo-patient, Pharmacist, Pharmacy assistant, Inappropriate, Illegal

## Abstract

**Background:**

Inappropriate over-the-counter supply of antibiotics in pharmacies for common infections is recognised as a source of antibiotic misuse that can worsen the global burden of antibiotic resistance.

**Objectives:**

To assess responses of community pharmacy staff to pseudo-patients presenting with symptoms of common infections and factors associated with such behaviour.

**Methods:**

A cross-sectional pseudo-patient study was conducted from Jan-Sept 2017 among 242 community pharmacies in Sri Lanka. Each pharmacy was visited by one trained pseudo-patient who pretended to have a relative with clinical symptoms of one of four randomly selected clinical scenarios of common infections (three viral infections: acute sore throat, common cold, acute diarrhoea) and a bacterial uncomplicated urinary tract infection. Pseudo-patients requested an unspecified medicine for their condition. Interactions between the attending pharmacy staff and the pseudo-patients were audio recorded (with prior permission). Interaction data were also entered into a data collection form immediately after each visit.

**Results:**

In 41% (99/242) of the interactions, an antibiotic was supplied illegally without a prescription. Of these, 66% (*n* = 65) were inappropriately given for the viral infections. Antibiotics were provided for 55% of the urinary tract infections, 50% of the acute diarrhoea, 42% of the sore throat and 15% of the common cold cases. Patient history was obtained in less than a quarter of the interactions. In 18% (44/242) of the interactions staff recommended the pseudo-patient to visit a physician, however, in 25% (11/44) of these interactions an antibiotic was still dispensed. Pharmacy staff advised the pseudo-patient on how to take (in 60% of the interactions where an antibiotic was supplied), when to take (47%) and when to stop (22%) the antibiotics supplied. Availability of a pharmacist reduced the likelihood of unlawful antibiotic supply (OR = 0.53, 95% CI: 0.31–0.89; *P* = 0.016) but not appropriate practice.

**Conclusions:**

Illegal and inappropriate dispensing of antibiotics was evident in the participating community pharmacies. This may be a public health threat to Sri Lanka and beyond. Strategies to improve the appropriate dispensing practice of antibiotics among community pharmacies should be considered seriously.

## Background

Medicines use is appropriate (rational and correct) when patients receive medicines appropriate to their clinical needs, in doses that meet their individual requirements, for an adequate period of time, and at affordable prices [1]. If any one of these conditions is not met, then it is referred to as inappropriate (irrational or incorrect) use of the medicines. It has been estimated that worldwide more than half of medicines are prescribed, dispensed or sold inappropriately [2, 3].

Inappropriate use of antibiotics is a global problem, particularly in the Asian region [4, 5]. It is common to see antibiotics provided inappropriately for self-limiting viral infections such as upper respiratory tract infections (URTIs) [5–8] and acute diarrhoea [6, 9], as well as bacterial infections including urinary tract infections (UTIs) [6, 10]. Inappropriate prescribing of antibiotics is observed in many developing countries [11] and though most of the URTIs are viral infections [12], there appears to be a high prevalence of antibiotic prescriptions provided for viral URTIs in developing and transitional countries, ranging from about 40 to 75% and for acute diarrhoea from about 20 to 55% [11]. A recent country-specific analysis reported a high rate of antibiotic use for viral URTIs in public primary care facilities in South East Asian countries, including Bangladesh (59% of viral URTIs were being treated with antibiotics); Bhutan (34%); Korea (65%); Rajasthan, India (94%); Karnataka, India (70%); Indonesia (72%); Maldives (43%); Myanmar (87%); Sri Lanka (70%); Thailand (43%) and East Timor (55%) [5].

Self-medication with antibiotics is also a major contributory factor to inappropriate use of antibiotics in the community [13]. The emergence and spread of antibiotic resistance (ABR), especially the appearance of multidrug-resistant bacterial strains which are highly resistant to many antibiotic classes, has raised a major global public health concern [14] and has been linked to the inappropriate use of antibiotics [15–17]. ABR is also associated with increased morbidity, mortality and treatment costs [18, 19] and the greatest burden occurs in low and middle-income countries (LMICs) [19]. If no actions are taken, it has been estimated that antimicrobial resistance will lead to 10 million deaths by 2050, and a loss of US$100 trillion of the world economic output [20–22].

A systematic review of nine surveys conducted in the Asian region, found that self-medication with non-prescription antimicrobials among the general public was 58% (7761 out of 13,366 of weighted cases) [16]. Studies have found that the main source of antibiotics used for self-medication is community pharmacies (CPs) [6, 16, 23, 24]. In China, Ye et al. reported that about 80% of the public purchased antibiotics without a prescription from CPs for self-medication [23]. In LMICs, the preferred method for purchasing medicines is through private pharmacies and often without a prescription. In Bangladesh, the public with a low income identified CPs as an important source of healthcare for all common health problems [25]. As in most LMICs, CPs or drug stores are usually a patient’s first point of contact with the healthcare system for advice on common ailments and other health problems [26]. The main reasons for this include, but are not limited to, patients’ inability to pay for both physician consultation fee and the prescribed medicine(s), limited time to visit a physician, and pharmacy specific factors, such as ease of access, long opening hours, the ability to purchase medicines in small quantities, credit facilities and personal familiarity and relationship with the pharmacist [27–29]. The people to physician ratio in most of the LMICs is lower than the 2010 WHO recommended ratio of 400:1 [30] and could also be one of the factors for people visiting pharmacies as a first point of contact with a healthcare professional.

Therefore, community pharmacists, being the first healthcare professional most people in LMICs approach for medical advice, such as common viral infections, are in the best position to help people with appropriate use of medicines. Pharmacists have the antibiotics knowledge necessary to ensure rational use of antibiotics [31] and can contribute to reducing ABR in the community. They can also contribute to the appropriate and safe use of antibiotics by providing advice to patients on antibiotics supply for prescription. In addition, pharmacists can play an important role in managing common infections by providing appropriate over-the-counter (OTC) medicines and non-pharmacological treatments, and referring patients to a medical practitioner, when necessary.

However in many LMICs, community pharmacists are selling antibiotics inappropriately for self-limiting viral URTIs [32–35], acute diarrhoea [32, 35, 36] and uncomplicated UTIs [32, 34]. Concerns have been raised about such inappropriate antibiotic dispensing practice due to profit aspirations, low quality of practice, insufficient drug sellers’ knowledge and training [28, 35, 37, 38]. Whilst anecdotally, there is evidence for supply of antibiotics without a prescription in Sri Lanka, there is very little empirical research on the provision of antibiotics in Sri Lankan community pharmacies. Therefore, this study aimed to determine community pharmacy staff’s (pharmacist or any other staff who attended to the pseudo-patient) responses when a pseudo-patient presented with symptoms of common infections and possible factors associated with such behaviour.

## Methods

### Study design

This pseudo-patient study was part of a larger study conducted among Sri Lankan CPs from January to September 2017. There were two arms to this study; one of which involved pseudo-patients’ direct antibiotic product requests (DPR) from 242 CPs throughout Sri Lanka [39]. The current findings were from the second arm, which involved pseudo-patient visits to the same 242 pharmacies but presenting with the clinical symptoms of one of four scenarios of common infections (symptoms-based requests- SBRs) including, acute sore throat (adult female), common cold (four year-old child), acute diarrhoea (adult male) and UTI (adult female). The DPR and SBR visits were conducted randomly within a time interval of approximately two to six weeks apart.

The pseudo-patient approach can be considered as a robust methodological tool for pharmacy practice research, especially as the knowledge of being observed can lead to behavioural change [40, 41]. Despite its own methodological disadvantages, in general, the pseudo-patient method increases the validity of the study design and accuracy of the findings compared to other self-reported qualitative or quantitative surveys mainly because of the absence of social-desirability bias [42, 43].

### Sample size calculation and sampling

The sample size for this study was derived from a previous phase: a self-reported cross-sectional country survey conducted among CP staff in Sri Lanka. The survey sample size (*n* = 369) was calculated based on the results of a previous pilot study (Zawahir S, Amarasinghe M, Hassali MA, Lekamwasam S: Knowledge, attitudes and practices related to antibiotic use among community and hospital pharmacists in district galle, Sri Lanka, Preparation) and the sample size calculation has been detailed in a previous publication [39]. A total of 267 (72%) pharmacies agreed to participate in the self-reported survey and all agreed to be approached to obtain consent for pseudo-patient visits and audio recordings of the visits. However, 243 pharmacies agreed to participate in the pseudo-patient visits and eventually 242 visits were made as one pharmacy went out of the business during the study. A total of 204 agreed to an audio recording of the interaction during the visit.

### Clinical scenarios and data collection

The scenarios were developed based on previously published literature [32, 44]. The scenarios and expected visit outcomes are detailed in Table 1. The pseudo-patients with the symptoms of viral infections were expected to be appropriately advised and provided with suitable OTC medicines (if necessary) and the pseudo-patients with uncomplicated UTI symptoms were expected to be referred to a physician.

**Table 1:** Detailed scenarios with rationale and expected outcome

Case	Reported symptoms	Additional information (If requested)	Rationale	Expected outcome
1	Pseudo-patient’s sister (25 years old) is having difficulty swallowing; it is painful when swallowing. She has a slight fever too. She has had symptoms for past three days. Requested some medicine to relieve her symptoms.	1. No known allergies. 2. No concurrent medicine. 3. No co-morbidities. 4. Gargled with salt water but didn’t help much. 5. Not tried any medicine. 6. No cough. 7. No headache. 8. Not visited a physician. 9. Not pregnant. 10. Not breast feeding.	URTIs are common self-limiting viral infections for which antibiotics are widely prescribed in Sri Lanka [5].	No antibiotic should be dispensed. The pseudo-patient should be advised to gargle with salt water; provide an OTC antipyretic e.g. paracetamol, for the fever. Advice on proper dose. The pseudo-patient should be advised to see the physician if symptoms continue for more than a week or get worse.
2	The antibiotic is for pseudo-patient’s niece (4 years old). She has been suffering from a productive cough, runny nose (clear mucus), slight fever, occasional sneezing and some loss of appetite. The symptoms started three days ago. Requested medicine to relieve the condition.	1. No known allergies. 2. No concurrent medicine. 3. No co-morbidities. 4. Tried chlorpheniramine maleate and paracetamol. 5. No difficulties in breathing. 6. No sore throat. 7. Clear nasal discharge. 8. No headache. 9. 1–2 coughs per hour. 10. Not visited a physician. 11. Brings up a little phlegm when she coughs. 12. The cough is not worse at night.	URTIs are common self-limiting viral infections for which antibiotics are widely prescribed in Sri Lanka [5].	No antibiotic should be dispensed. The pseudo-patient should be advised to use paracetamol for fever. Advice on proper dose. Advice to see the physician if symptoms continue for more than a week, or they get worse (in particular fever and aches).
3	The antibiotic is for pseudo-patient’s younger brother (20 years old) who is having acute loose bowel motion for the past two days (watery diarrhoea). He has to go to toilet almost every 3–4 h. The pseudo-patient requested some medicine to alleviate the reported symptoms.	1. No known allergies. 2. No concurrent medicine. 3. No co-morbidities. 4. Tried diphenoxylate hydrochloride, it has helped a little but still has watery diarrhoea and going to toilet every 3–4 h. 5. Taking oral rehydration solution as well. 6. No vomiting. 7. No mucus or blood in stools. 8. No abdominal pain. 9. No appetite. 10. Not visited a physician. 11. No fever. 12. Currently, no family member is having similar symptoms.	Acute respiratory infections, diarrhoea, and neonatal infections remain major problems particularly in children in South Asian countries [56].	No antibiotic should be dispensed. Advice to take Oral rehydration solution. Proper Oral rehydration solution preparation method should be discussed. Hygiene advice should be provided such as hand washing. The pseudo-patient should be advised to see a physician, if the diarrhoea continues for a week or gets worse.
4	The antibiotic request is for pseudo-patient herself. Reported symptoms are discomfort on urination with a burning sensation and the need to urinate more frequently. She has been drinking more water than usual to alleviate the symptoms. She also has a slight fever. The symptoms started two days ago. Requested some medicine to cure the reported symptoms.	1. No known allergies. 2. No concurrent medicine. 3. No comorbidities. 4. Not tried anything. 5. Low grade fever. 6. No back pain. 7. No genital ulcer. 8. She is not pregnant/not expecting to be pregnant in near future. 9. Not visited a physician. 10. Last time had the same problem about 12 months ago	Approximately 50% of women are treated for UTIs with antibiotics at some point in their lifetime [57].	No antibiotic should be dispensed. The pseudo-patient should be advised to see a physician.

OTC- Over the counter; URTIs- Upper respiratory tract infections; UTIs- Urinary tract infections

Thirty-two pseudo-patients were involved in the visits. They were either recent pharmacy graduates or pharmacy students from two public universities. The research assistants who were involved in obtaining informed consent, did not participate in the pseudo-patient visits. Each of the participating pharmacies was visited by a pseudo-patient and a research assistant. While the pseudo-patient interacted with the pharmacy staff, the accompanying research assistant observed and covertly audio recorded the interaction during the visits. Each pseudo-patient requested an unspecified medicine for the treatment of the symptoms of one of four randomly selected clinical scenarios of common infections (acute sore throat, common cold, acute diarrhoea (possible viral infections), and a bacterial uncomplicated UTI). Three levels of requests were made by the pseudo-patient to obtain an antibiotic. The first level of request consisted of requesting an unspecified medicine to alleviate the reported symptoms of the common infection. If an antibiotic was not given, the pseudo-patient used the second level of the request; “Can’t you give me something stronger?” If the pharmacy staff did not provide an antibiotic, the pseudo-patient openly stated, “I would like an antibiotic,” which was considered as the third level of request. If the pharmacy staff asked any questions related to reported symptoms, pseudo-patients were trained to answer according to the pre-determined scenarios.

In addition, advice provided by pharmacy staff and the availability of a pharmacist during the visit were noted. The availability of a pharmacist was confirmed as follows, a research assistant observed the pharmacy licence displayed in the pharmacy with a photograph of the pharmacist. If the photo displayed did not match the attending pharmacy staff or there was no photo displayed, then the pseudo-patient asked “Can I talk to your pharmacist, please?” The availability of the pharmacist was then based on the response to this question. In Sri Lanka, the licence issued by the National Medicine Regulatory Authority to run a community pharmacy should be displayed in the pharmacy with the photo of a pharmacist who owns the pharmacy or is employed [45].

Although as part of the visit the pseudo-patient did not ask why an antibiotic was not provided, any reason stated spontaneously by the pharmacy staff was captured from the audio-recording and reported accordingly.

Immediately after each visit, the pseudo-patient and research assistant completed the data collection sheet (Table 2) together while listening to the audio recording. The questions in the data collection sheet were based on WWHAM (Who for, What symptoms, How long, Any medicine tried, other Medication taken) [46] and What-Stop-Go [47] protocols.

**Table 2:** Information included in the data collection sheet

	Data collected
1	Geographical location of the pharmacy
2	Details of attending pharmacy staff
4	Requested a prescription
4	Whether antibiotic dispensed
5	Antibiotic dispensing detail (level of request, type, dose and frequency)
6	WWHAMM questions Who is the medicine for? What are the symptoms? How long have you had the symptoms? What action has already been taken? Are you taking any other medicine? Have other medical and lifestyle history taken? (specific to the scenario)
7	Other medical and lifestyle history inquired by pharmacy staff Age, gender, Allergies, Environmental exposure, Suspected adverse drug reaction and any other related to specific scenario
8	Patient advice on dispensing Including how much to take, how to take, when to take, how often to take and when to stop.
9	Recommendations including provision of OTC medicine and referrals to a physician

### Data analysis

Descriptive statistics such as frequencies (%) were used to describe the data. Pearson’s chi-square test and binary logistic regression analysis were performed using independent predictors (availability of pharmacist, gender, geographical area of the pharmacy, type of scenario presented and type of pharmacy) to evaluate the possible factors associated with antibiotic supply without a prescription for reported common infections. The *P* value of < 0.05 was considered as statistically significant. SPSS version 24 was used for all the analyses.

## Results

A total of 242 pharmacies were visited by the pseudo-patients. The types of pharmacies which agreed to the pseudo-patient visits included, private chain pharmacies (45%; 109/242), private single pharmacies (43.8%; 106/242), semi-government pharmacies (7%; 17/242) and pharmacies in private hospitals (4.1%; 10/242). The clinical scenario of uncomplicated UTI of adult female was presented to 62 CPs and the other three scenarios - acute sore throat (adult), common cold (four-year-old child) and acute diarrhoea (adult), were presented equally among 180 pharmacies.

**Fig. 1:**
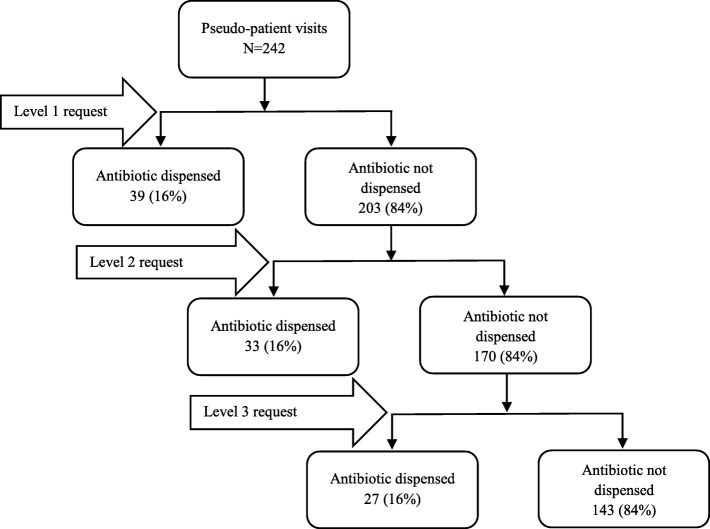
Levels of antibiotic requests and dispensing. Level 1 request – Requesting an unspecified medicine to alleviate the reported symptoms of the common infection. Level 2 request – “Can’t you give me something stronger?”. Level 3 request – “I would like an antibiotic”

Overall, in 41% (99/242) of instances, antibiotics were sold illegally without a prescription in response to the pseudo-patients reported clinical symptoms (Table 3). The adults’ pseudo-patient scenarios of acute sore throat, acute diarrhoea, and uncomplicated UTI accounted for the highest proportions of illegal antibiotic sales (49%; 90/182), whereas pharmacy staff were more reluctant to sell antibiotics without a prescription when pseudo-patients presented with the symptoms of the common cold for a child (15%; 9/60). The adult common infection scenarios were significantly more likely to receive antibiotics compared to the paediatric one, χ^2^ (1, *N* = 242) = 22.15, *P* < 0.001). In two-thirds of the instances antibiotics were sold inappropriately for underlying viral infections (65/99) including acute sore throat, common cold and acute diarrhoea. In the majority of instances an antibiotic was sold upon the 1st or 2nd level of request (73%; 72/99) without the pseudo-patient requesting an antibiotic by name, and the rest were supplied on the 3rd level of request (Fig. 1). About half of the visited pharmacies were observed to have a pharmacist on duty. In about two-thirds of the instances (61%; 60/99) antibiotics were sold by a pharmacy staff member other than a qualified pharmacist. Though availability of a pharmacist significantly reduced the likelihood of antibiotics supply without a prescription (OR = 0.53, 95% CI: 0.31 to 0.89; *P* = 0.016), that was not impacted on antibiotic supply between viral and bacterial infections (OR = 1.02, 95% CI: 0.41 to 2.53; *P* = 0.972).

**Table 3:** Antibiotic sale without a prescription based on reported clinical case

All cases	Pseudo-patient case presented, frequency (%)				
	Overall *n* = 242	Sore throat *n* = 60	Common cold *n* = 60	Diarrhoea *n* = 60	UTI *n* = 62
1st level of request (Can I get some medicine to alleviate the symptoms)	39 (16)	11 (18)	1 (2)	9 (15)	18 (29)
2nd level of request (Can I get something stronger)	33 (14)	7 (12)	6 (10)	11 (18)	9 (15)
3rd level of request (I would like an antibiotic)	27 (11)	8 (13)	2 (3)	10 (17)	7 (11)
Antibiotic dispensed (all degree)	99 (41)	26 (43)	9 (15)	30 (50)	34 (55)
Antibiotic not dispensed	143 (59)	34 (57)	51 (85)	30 (50)	28 (45)
Antibiotics dispensed cases	*n* = 99	*n* = 26	*n* = 9	*n* = 29	*n* = 34
Ciprofloxacin	29 (30)	1 (4)	Nil	2 (7)	26 (76)
Metronidazole	23 (23)	Nil	Nil	23 (79)	Nil
Erythromycin	19 (20)	17 (65)	Nil	2 (7)	Nil
Amoxicillin	9 (9)	1 (4)	8 (89)	Nil	Nil
Azithromycin	8 (8)	7 (27)	Nil	1 (3)	Nil
Norfloxacin	5 (5)	Nil	Nil	Nil	5 (15)
Other antibiotics	7 (4)	Nil	1 (11)	1 (3)	3 (9)

Overall, only a few pharmacy staff asked the pseudo-patients about concurrent medical conditions (10%; 25/242), any action that has already been taken (8%; 20/242) and concurrent medicines used (1.7%; 4/242). In 18% (44/242) of the instances, pseudo-patients were recommended to see a physician. However, in about a quarter of them (25%; 11/44) an antibiotic was still provided. Only a quarter of the pseudo-patients with UTI (24%; 15/62) were advised to see a physician (Table 4).

**Table 4:** Patient history taking, counselling and recommendation

	Frequency (%)				
	Overall *n* = 242	Sore throat *n* = 60	Common cold *n* = 60	Diarrhoea *n* = 60	UTI *n* = 62
Asked about other symptoms	25 (10)	7 (12)	11 (18)	6 (10)	2 (2)
Action has been taken	20 (8)	5 (8)	9 (15)	4 (7)	2 (3)
Taking any other medicine	4 (1.7)	2 (3)	1 (2)	0	1 (2)
Recommended to see a physician	44 (18)	6 (10)	14 (23)	9 (15)	15 (24)
Antibiotic dispensed cases, frequency (%)					
Questions asked about;	*n* = 99	*n* = 26	*n* = 9	*n* = 30	*n* = 34
Other symptoms/ comorbidities (Yes)	36 (36)	10 (38)	6 (67)	14 (47)	6 (18)
Action already taken	12 (12)	3 (11)	3 (33)	4 (13)	2 (6)
Other medicine taking	2 (2)	1 (4)	0	0	1 (3)
Pregnancy status	0	N/A	N/A	N/A	0
Drug allergies	10 (10)	5 (19)	0	2 (7)	3 (9)
Patient counselling/ advice;					
Recommended to see a physician	10 (10)	2 (8)	0	5 (17)	3 (9)
How to take	59 (60)	16 (62)	3 (33)	16 (53)	24 (71)
How often to take	47 (47)	10 (38)	3 (33)	13 (43)	21 (62)
When to stop taking	22 (22)	4 (15)	0	7 (23)	11 (32)

*N/A* Not applicable

In about one-third of the pharmacies (36%; 36/99) where an antibiotic was sold, the pseudo-patients were further questioned about their symptoms or concurrent medical conditions. The questions related to action that has already been taken (12%; 12/99), drug allergies (10%; 10/99), and concurrent medicines used (2%; 2/99). About half of the pseudo-patients were advised on how and how often to take the provided antibiotic, and about a quarter of them were advised on when to stop taking the antibiotic. Availability of a pharmacist in the pharmacy had no impact on patient counselling. In none of the pharmacies did staff inquire about the pregnancy status of the pseudo-patients when selling an antibiotic for a childbearing-aged female pseudo-patient presenting with symptoms of UTI (Table 4). None of the pseudo-patients with diarrhoea were questioned about important symptoms, such as, the presence of blood in stool, fever, prolonged episodes of watery stools, or significant complications of diarrhoea such as dehydration and vomiting. The median interaction time between the pseudo-patients who received an antibiotic and pharmacy staff was 2 min (IQR = 1–3 min).

The most common antibiotic sold to pseudo-patients with sore throat was erythromycin (65%; 17/26), amoxicillin for common cold (89%; 8/9), metronidazole for acute diarrhoea (79%; 23/29) and ciprofloxacin for female UTI cases (77%; 26/34) (Table 3).

In 143 pharmacies, the staff did not provide an antibiotic to the pseudo-patient. The primary reason for not supplying an antibiotic was the absence of a prescription from a physician (100/143; 70%). None of the pharmacy staff discussed the reported symptoms with the pseudo-patient. They did not discuss issues such as severity of the current health condition, possible aetiology of the infection, risk of emergence of ABR if an antibiotic is inappropriately supplied for a viral infection and risk of supplying an antibiotic for a possible bacterial infection (UTI) without being diagnosed by a physician. Instead, they simply denied giving an antibiotic with or without stating a reason. The median interaction time between the pseudo-patient who did not receive an antibiotic and pharmacy staff was also 2 min (IQR = 1–3 min).

## Discussion

To the best of our knowledge, this is one of the first two pseudo-patient studies conducted in Sri Lanka, including all different types of community pharmacies throughout the country, to evaluate pharmacy staff’s behaviour when presented with symptoms of common infections.

Despite Sri Lankan laws explicitly prohibiting the supply of any antibiotic without a prescription, regardless of the patient’s medical condition or symptoms, this study found that antibiotics were not only commonly provided without a prescription (illegal) for common infections, but also inappropriately for viral infections. The antibiotics were supplied without even being specifically requested by the pseudo-patients. In addition, pharmacy staff failed to adequately inquire about the presenting symptoms, give correct advice or offer alternative OTC products. However, the overall supply was lower when the presenting common infection was that of a child’s, and when a pharmacist was present.

The current results showed that unlawful antibiotic supply was high (41%) and this finding was supported by DPR pseudo-patient visit findings from the same pharmacies, where antibiotics were provided without a prescription to pseudo-patients in 61% of the interactions [39]. The major reason for the prevailing situation with regard to unlawful antibiotic supply among community pharmacies in Sri Lanka may be poor regulation of antibiotics supply in the country and this has been discussed in the DPR arm of the study [39]. A similar poor regulation of medication-dispensing policies has also accounted for variable rates of non-prescription antibiotic sales in other parts of the world [16, 27, 32, 40, 48].

This study also revealed inappropriate supply of antibiotics in response to reported symptoms of common infections of viral aetiology. As the pseudo-patient clinical scenarios were representing possible viral infections (acute sore throat, common cold and acute diarrhoea) and a probable bacterial infection (uncomplicated UTI), the expected behaviour of the staff for the reported scenarios was to effectively obtain relevant medical and lifestyle-related history, advise the pseudo-patient appropriately, provide an OTC medicine or non-pharmacological treatment (as necessary, for viral infections) or refer them to a physician (in the case of UTI). Despite this fact about two thirds of staff who gave out an antibiotic, had supplied them inappropriately for viral infections. The potential reasons for such behaviour of community pharmacy staff have been discussed in a recent self-reported national survey conducted among community pharmacy staff in Sri Lanka which mainly highlighted staff’s inadequate clinical experience and knowledge about antibiotics (Zawahir S, Lekamwasam S, Aslani P: A cross-sectional national survey of community pharmacy staff: knowledge and antibiotic provision, submitted). Similar behaviour has also been observed in many other LMICs [16, 32, 35, 49]. A pseudo-patient study conducted in Riyadh, Saudi Arabia found that irrespective of the aetiology of the infections, antibiotics were freely dispensed without a prescription for all the presented clinical symptoms of sore throat, acute sinusitis, otitis media, acute bronchitis, diarrhoea and UTI [32]. Ayele *at el.,* found that community pharmacy staff in Northwest Ethiopia dispensed antibiotics inappropriately for self-limiting acute diarrhoea and URTIs [35]. Illegal and inappropriate supply of antibiotics in pharmacies will not only promote ABR but also be associated with significant adverse events including drug side effects, high medical costs, and complications of infections leading to longer hospital stays and possible emergence of multi drug resistance.

When comparing the findings of the DPR arm of the study [39] to the current SBR study findings, it can be seen that a large proportion of the pharmacies supplied antibiotics illegally, on both occasions when visited by the pseudo-patient. This demonstrates that, potentially, the same reasons explain the provision of antibiotics without a prescription, whether the pseudo-patient requests an antibiotic by name [39] or presents with symptoms of a common infection which the pharmacy staff believe can be treated by an antibiotic. Therefore, there is substantial room for practice improvement, both in increasing clinical knowledge as well as enforcing the legal requirements surrounding antibiotics supply. However, the current study found that the proportion of community pharmacies providing an antibiotic without a prescription was 20% less when there was a SBR compared to a DPR. This difference may be due to several reasons. The observed higher prevalence of antibiotic supply during DPR may be due to the staff’s false perception that when the patient is requesting an antibiotic by a specific product name, the patient has knowledge about it or has had previous experience in using it. They may therefore feel more confident in providing an antibiotic. In the case of SBR, the pseudo-patient was required to describe the symptoms to the pharmacy staff, which may have initiated more discussion and an increased effort from the staff to appropriately diagnose and provide treatment options other than an antibiotic. Furthermore, the staff may have felt that the pseudo-patient had not tried any products in the past, and so they may have been less confident in providing an antibiotic.

The observed low proportion of antibiotic supply (15%) for the reported paediatric scenario is a positive sign. This could be due to either pharmacy staff’s concern about vulnerable paediatric patients or their lack of clinical competency in dealing with such patients, or perhaps both. As none of the staff determined the cause of the infection and failed to educate the pseudo-patient appropriately whether an antibiotic was needed or not, also supports the argument above about limited clinical training and therefore knowledge of staff.

Further, only about half of the pseudo-patients who obtained antibiotics, received any form of counselling or advice. Counselling patients on “when to stop taking antibiotics” does not appear to be part of the current process of providing antibiotics in Sri Lankan pharmacies. A similar inadequate patient history taking and lack of patient counselling was also observed in the DPR arm of the pseudo-patient study [39]. This is not only a problem in Sri Lanka. Other studies from LMICs have also highlighted similar issues [38, 50]. Pharmacy staff’s beliefs about the usefulness of counselling, time constraints, absence of any patient counselling guidelines in Sri Lanka and/or lack of privacy in community pharmacies and limited clinical knowledge, may have contributed to the poor counselling observed. This provides an important opportunity for continued professional development of Sri Lanka pharmacy staff.

Although it was revealed that the presence of a pharmacist in the pharmacy may have been associated with a lower likelihood of antibiotic supply without a prescription, the presence of the pharmacists did not impact the counselling received by the pseudo-patient nor result in an appropriate response to the reported symptoms of common infections. Therefore, this supports the argument above about limited clinical training and the knowledge of staff. It is also evident from the literature that un-qualified pharmacy staff or pharmacists with poor clinical knowledge may be contributing to inappropriate antibiotic supply [39, 51].

The inappropriate provision of antibiotics and inadequate counselling provided to pseudo-patients observed in this study challenges the goal of appropriate use of antibiotics in communities, and can contribute to global antibiotic misuse [52]. In turn, this can have a serious public health threat through contributing to antibiotic resistance at individual and population levels [53]. Therefore, the prevailing situation related to illegal and inappropriate antibiotic supply in Sri Lanka is not only challenging to the public health of the country, but has global consequences [54].

## Limitations

Although repeated training and rehearsals were made to ensure consistency between pseudo-patients and to increase the internal validity of the data collected, it is still possible that some interpersonal differences among pseudo-patients may have impacted the behaviour of the pharmacy staff. This approach may also have limited external validity, since in normal circumstances the pharmacist would probably have much more information about the client. A real patient has the tendency to communicate freely about his/her pathology, therefore, the outcomes measured by this method may vary from real situations [55]. Furthermore, self-selection of the study participants may have impacted the study findings.

## Conclusions

It is evident from this study that antibiotics are given out from pharmacies illegally without a prescription and clinically inappropriately. Presence of a pharmacist in the pharmacy may have reduced the illegal supply but it did not appear to impact appropriate practice.

Immediate action is sought from all stakeholders including healthcare professionals, local policy makers as well as global agencies such as WHO, and the public, to curb this public health issue. In addition to strict implementation of policies, awareness and educational interventions must be implemented to improve appropriate antibiotic dispensing practice among pharmacists and their staff.

## Abbreviations

ABR: Antibiotic resistance
CI: Confidence interval
CP: Community pharmacy
DPR: Direct product request
LMIC: Low and middle-income countries
OR: Odds ratio
OTC: Over the counter
SBR: Symptoms based request
URTIs: Upper respiratory tract infections
UTI(s): Urinary tract infection(s)
WWHAM: Who for, What symptoms, How long, Any medicine tried, other Medication taken
